# Chassis engineering of *Escherichia coli* for *trans*‐4‐hydroxy‐l‐proline production

**DOI:** 10.1111/1751-7915.13573

**Published:** 2020-05-12

**Authors:** Xiulai Chen, Juyang Yi, Wei Song, Jia Liu, Qiuling Luo, Liming Liu

**Affiliations:** ^1^ State Key Laboratory of Food Science and Technology Jiangnan University Wuxi 214122 China; ^2^ Key Laboratory of Industrial Biotechnology Ministry of Education Jiangnan University Wuxi 214122 China; ^3^ National Engineering Laboratory for Cereal Fermentation Technology Jiangnan University Wuxi 214122 China; ^4^ Shaoxing Baiyin Biotechnology Co. Ltd Shaoxing 312000 China

## Abstract

Microbial production of *trans*‐4‐hydroxy‐l‐proline (Hyp) offers significant advantages over conventional chemical extraction. However, it is still challenging for industrial production of Hyp due to its low production efficiency. Here, chassis engineering was used for tailoring *Escherichia coli* cellular metabolism to enhance enzymatic production of Hyp. Specifically, four proline 4‐hydroxylases (P4H) were selected to convert l‐proline to Hyp, and the recombinant strain overexpressing *Ds*P4H produced 32.5 g l^−1^ Hyp with α‐ketoglutarate addition. To produce Hyp without α‐ketoglutarate addition, α‐ketoglutarate supply was enhanced by rewiring the TCA cycle and l‐proline degradation pathway, and oxygen transfer was improved by fine‐tuning heterologous haemoglobin expression. In a 5‐l fermenter, the engineered strain *E. coli*Δ*sucCD*Δ*putA*‐VHb_(L)_‐*Ds*P4H showed a significant increase in Hyp titre, conversion rate and productivity up to 49.8 g l^−1^, 87.4% and 1.38 g l^−1^ h^−1^ respectively. This strategy described here provides an efficient method for production of Hyp, and it has a great potential in industrial application.

## Graphical abstract

Microbial production of *trans*‐4‐hydroxy‐l‐proline (Hyp) offers significant advantages over conventional chemical extraction. Here, chassis engineering was used for tailoring *Escherichia coli* cellular metabolism to enhance enzymatic production of Hyp. In a 5‐l fermenter, the engineered strain showed a significant increase in Hyp titer, conversion rate, and productivity up to 49.8 g l^−1^, 87.4%, and 1.38 g l^−1^ h^−1^, respectively.

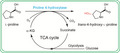

## Introduction

*trans*‐4‐Hydroxy‐l‐proline (Hyp), one of the hydroxyproline isomers, is a useful chiral building block in medicine, biochemistry, food, cosmetic and other aspects of industry (Yi *et al.*, 2014). Hyp is generally produced in industry by acid hydrolysis of animal collagen, which is a complex process with many bottleneck problems such as low efficiency and heavy environmental pollution (Zhao *et al.*, 2017). To overcome these problems, Hyp biosynthesis is regarded as a promising method due to its high catalytic efficiency and environmental compatibility (Shibasaki *et al.*, 2000b; Zhao *et al.*, 2017).

Recently, four metabolic engineering strategies have been developed to reconstruct an efficient cell factory for Hyp production, and they mainly relate to a key enzyme, proline 4‐hydroxylase (P4H) that can catalyse the hydroxylation of l‐proline to Hyp (Table 1). Strategy I is to screen Hyp‐producing strains. Some bacteria or fungi have been found to form Hyp via fermentation directly (Serizawa *et al.*, 1995). In addition, *Escherichia coli* NA45 was isolated from *E. coli* BL21/pUC19‐TTP‐P4H by chemo‐physical combination mutagenesis, which could convert l‐proline to Hyp with glycerol as a sole carbon source (Wang *et al.*, 2016). Strategy II is to select and express P4H. When the P4H gene from *Dactylosporangium* sp. RH1 (*Ds*P4H) was expressed in *E. coli* W1485Δ*putA*, *E. coli* BL21(DE3) and *Corynebacterium glutamicum* (Shibasaki *et al.*, 2000b; Yi *et al.*, 2014), the highest concentration of Hyp was up to 41 g l^−1^ with l‐proline and glucose as substrates (Shibasaki *et al.*, 2000b). In view of this, Hyp production from glucose was firstly achieved in an l‐proline‐producing *E. coli* by expressing *Ds*P4H (Shibasaki *et al.*, 2000a). Additionally, Hyp production (45.83 g l^−1^) was largely enhanced from glucose by expressing P4H from *Alteromonas mediterranea* and a γ‐glutamyl kinase (proB) mutation in *E. coli* MG1655Δ*putA* (Wang *et al.*, 2018). Strategy III is to introduce haemoglobin into *E. coli* for oxygen transfer. When the Vitreoscilla haemoglobin (VHb) gene was integrated into the chromosome of the *Ds*P4H‐expressing strain *E. coli* WD3(pTrc99a‐p4h), Hyp production (14.4 g l^−1^) was increased by 73.2% compared to that of *E. coli* strain without VHb (Zhao *et al.*, 2017). Strategy IV is to engineer Hyp biosynthetic pathway. Hyp titre (21.72 g l^−1^) was improved in an l‐proline‐producing *C. glutamicum* by expressing and optimizing *Ds*P4H, deleting succinyl‐CoA synthetase (SucCD) gene, and expressing feedback‐resistant proB* gene (Falcioni *et al.*, 2015; Zhang *et al.*, 2019). Similarly, the final Hyp concentration of 31.0 g l was obtained by overexpressing *Ds*P4H, proB and glutamate‐semialdehyde dehydrogenase (proA), and knocking out proline dehydrogenase (PutA), α‐ketoglutarate dehydrogenase (SucAB), isocitrate lyase (AceA) and isocitrate dehydrogenase kinase/phosphatase (AceK; Zhang *et al.*, 2018a). The above research results have indicated that Hyp production can be successfully improved by enzymatic transformation and microbial fermentation. However, the hydroxylation of proline is strongly interconnected with central carbon metabolism in host (Loenarz and Schofield, 2011; Falcioni *et al.*, 2013; Falcioni *et al.*, 2015), and thus many physiology‐related factors potentially can interfere with the catalytic performance.

**Table 1:** Comparison of Hyp production by the engineered microorganisms

Strains	Hyp titre (g l^−1^)	Conversion rate (%)	Productivity (g l^−1^ h^−1^)	References
*E. coli* NA45	25.4	38.1	0.59	Wang *et al.* (2016)
*E. coli* W1485 *putA/p*WFH1	41.0	87.0	0.41	Shibasaki *et al.* (2000b)
*E. coli* W1485 *putA/p*WFP1	25.0	–	0.25	Shibasaki *et al.* (2000a)
*E. coli* SEcH(pTc‐B74A‐alp4h)	45.83	–	1.27	Wang *et al.* (2018)
*E. coli* WD3‐VGB(pTrc99a‐p4h)	14.4	62.6	0.25	Zhao *et al.* (2017)
*C. glutamicum* Hyp‐7	21.72	–	0.36	Zhang *et al.* (2019)
*E. coli* 3∆W3110/pTrc99a‐p4hy‐proba	31.0	–	0.60	Zhang *et al.* (2018a)
*E. coli*Δ*sucCD*Δ*putA*‐VHb_(L)_‐*Ds*P4H	49.8	87.4	1.38	This study

**Fig. 1:**
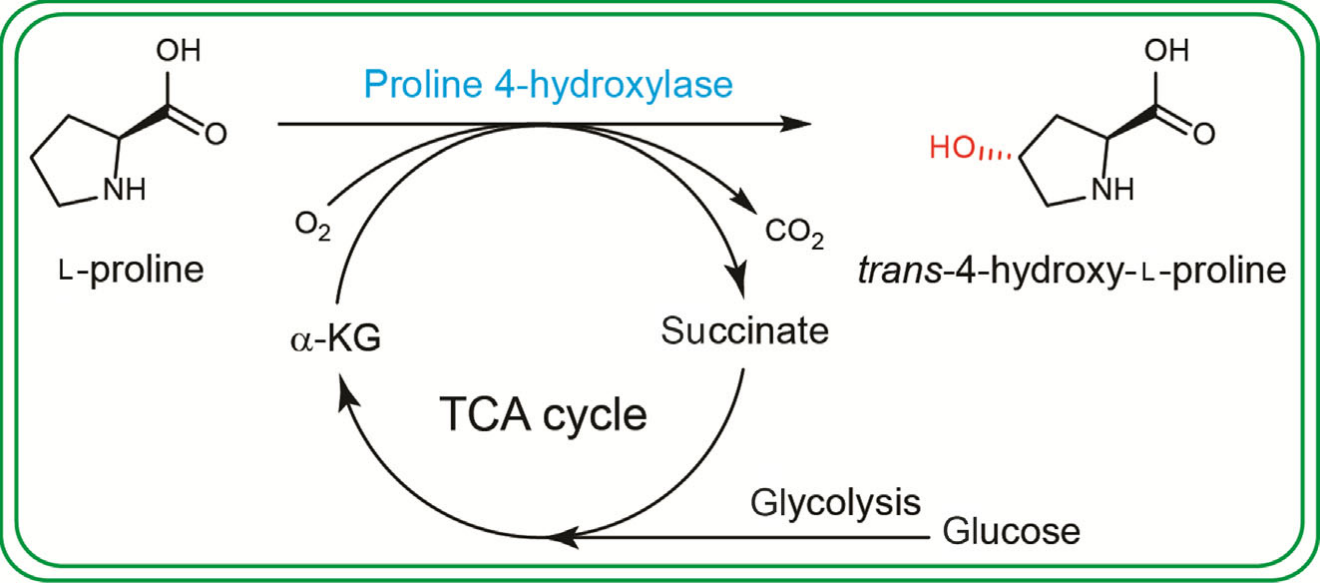
Chassis engineering of *E. coli* for enzymatic production of Hyp by combining proline 4‐hydroxylase with metabolic pathway. α‐KG, α‐ketoglutarate.

In this study, we described chassis engineering strategies to optimize the catalytic performance of *E. coli* for Hyp production*.* By combining central carbon metabolism with enzymatic transformation, its interconnection was established with α‐ketoglutarate (α‐KG; Fig. 1). Based on this interconnection, the whole‐cell biocatalysis was optimized systematically by metabolic engineering to enable an efficient production of Hyp. Under controlled culture conditions, the engineered strain *E. coli*Δ*sucCD*Δ*putA*‐VHb_(L)_‐*Ds*P4H produced up to 49.8 g l^−1^ Hyp in a 5‐l fermenter.

## Results

### Constructing the synthetic pathway for Hyp production

**Fig. 2:**
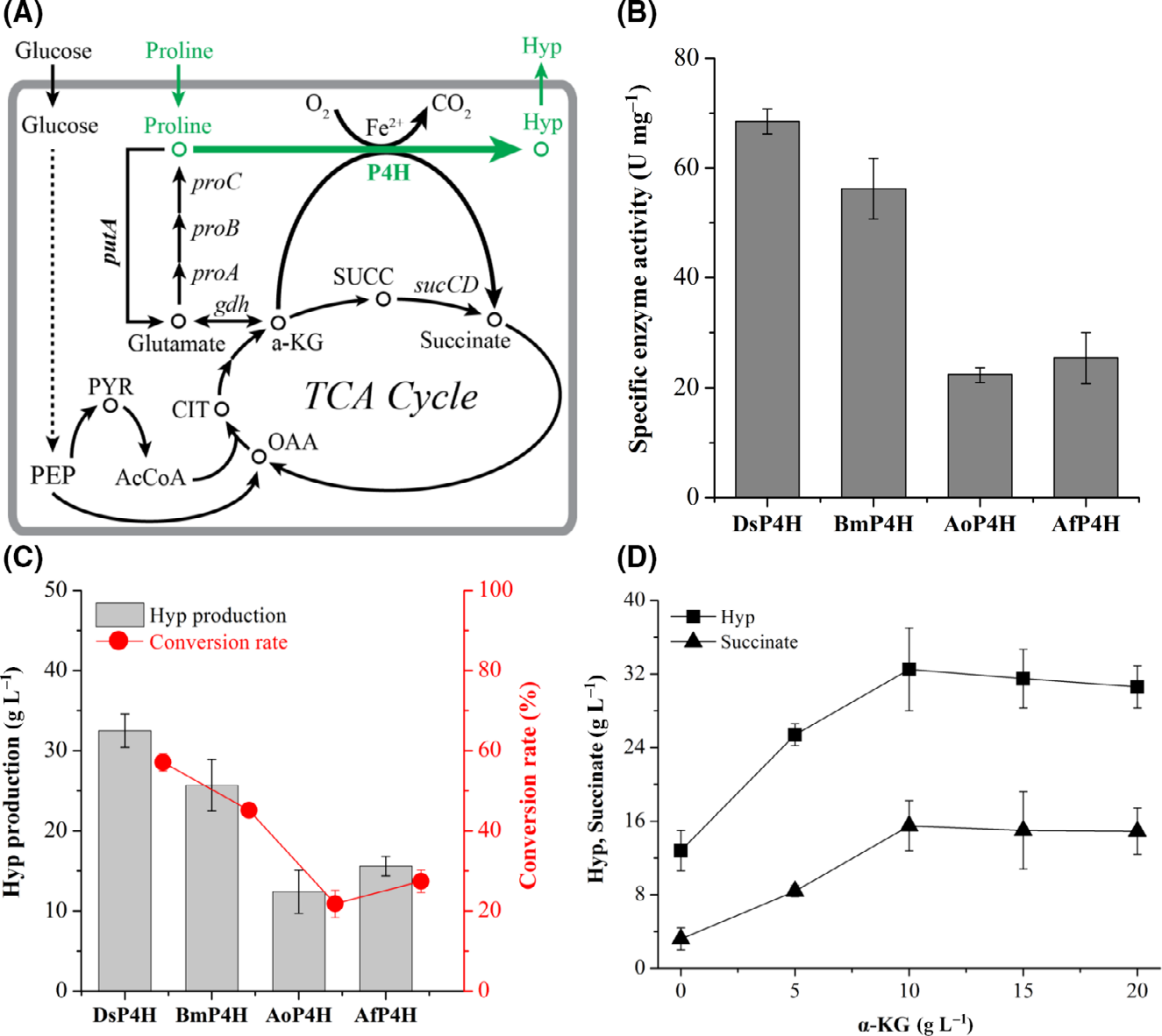
Constructing the synthetic pathway for Hyp production. A. The synthetic pathway for Hyp production with proline 4‐hydroxylase. B. The specific activities of four P4Hs in the corresponding recombinant strains respectively. C. Effect of *Ds*P4H, *Bm*P4H, *Ao*P4H and *Af*P4H on Hyp production and conversion rate respectively. D. Effect of α‐KG addition on Hyp and succinate production with strain *E. coli*‐*Ds*P4H. AcCoA, acetyl‐CoA; CIT, citrate; *gdh*, glutamate dehydrogenase gene; OAA, oxaloacetate; PEP, phosphoenolpyruvate; *proA*, glutamate‐semialdehyde dehydrogenase gene; *proB*, γ‐glutamyl kinase gene; *proC*, Δ^1^‐pyrroline‐5‐carboxylate reductase gene; PYR, pyruvate; SUCC, succinyl‐CoA.

Proline 4‐hydroxylase (P4H) can catalyse the hydroxylation of l‐proline at the 4‐position to produce *trans*‐4‐hydroxy‐l‐proline (Hyp) in the presence of α‐ketoglutarate (α‐KG), oxygen and ferrous ion (Lawrence *et al.*, 1996; Fig. 2A). P4H from *Dactylosporangium* sp. RH1 (*Ds*P4H) has been used for enzymatic production of Hyp with l‐proline as substrate (Shibasaki *et al.*, 2000b). To identify the superior enzyme, three P4Hs from *Bacillus megaterium* (*Bm*P4H), *Aspergillus oryzae* (*Ao*P4H) and *Aspergillus flavus* (*Af*P4H) were selected from Uniprot database with *Ds*P4H as a probe. Then, we cloned and overexpressed *Ds*P4H, *Bm*P4H, *Ao*P4H and *Af*P4H respectively. Next, the activities of four P4Hs were assayed, and they showed hydroxylation activities of 68.5, 56.2, 22.3 and 25.4 U mg^−1^ respectively (Fig. 2B). Finally, the effect of four P4Hs on Hyp production was investigated with 50 g l^−1^ l‐proline as substrate, and strain *E. coli*‐*Ds*P4H produced the highest concentration of Hyp up to 32.5 g l^−1^ with its conversion rate 57.1%, which was 26.5%, 162.1% and 108.3% higher than that of *E. coli*‐*Bm*P4H, *E. coli*‐*Ao*P4H and *E. coli*‐*Af*P4H respectively (Fig. 2C). Thus, the recombinant *E. coli*‐*Ds*P4H was selected for further research.

### Engineering the α‐KG‐supplying pathways to enhance Hyp production

*E. coli*‐*Ds*P4H could be used for efficiently converting l‐proline to Hyp with α‐KG addition. Thus, the effect of α‐KG addition on Hyp production was analysed with whole‐cell biocatalyst *E. coli*‐*Ds*P4H. Hyp titres were increased with α‐KG addition from 0 to 10 g l^−1^, and the maximal Hyp production was observed at 10 g l^−1^ α‐KG (Fig. 2D). When the addition of α‐KG was over 10 g l^−1^, Hyp production was slightly reduced (Fig. 2D). In this process, the main by‐product, succinate, was also measured during the enzymatic production of Hyp. With the increase of Hyp titres, succinate concentrations were increased gradually (Fig. 2D). When 10 g l^−1^ α‐KG was added, the maximal concentration of succinate was up to 15.5 g l^−1^, which was 3.8‐fold higher than that of no α‐KG addition (Fig. 2D). To sum up, conversion ratio with *E. coli*‐*Ds*P4H was up to 57.1% with α‐KG addition, but this conversion ratio was reduced to 22.5% without α‐KG addition. These results showed that additional supply of α‐KG was necessary for efficient production of Hyp, possibly due to the fact that *E. coli* host did not produce enough α‐KG for this transformation reaction.

**Fig. 3:**
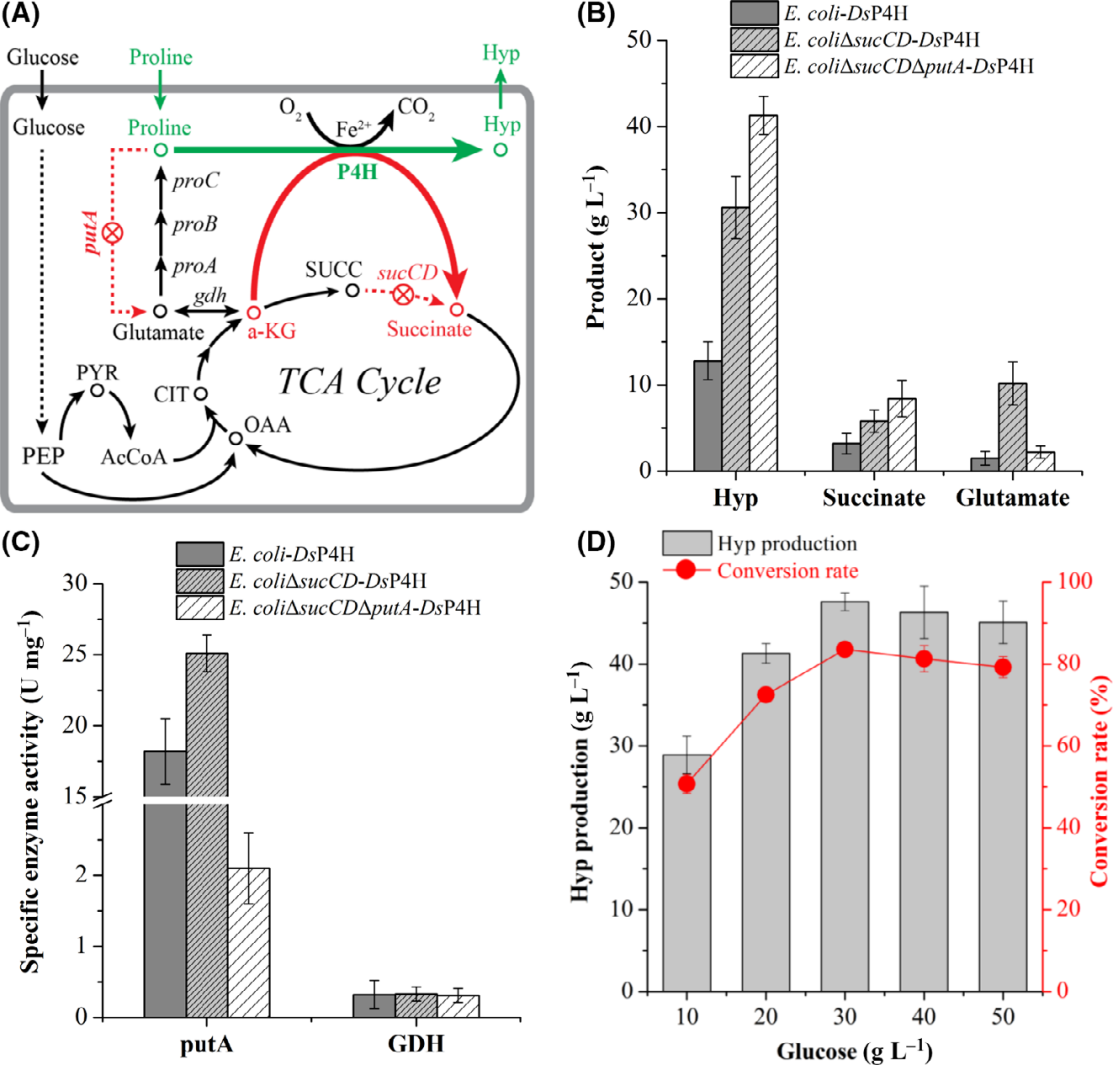
Engineering the α‐KG‐supplying pathways to enhance Hyp production. A. The α‐KG‐supplying pathways for enhancing Hyp production. B. Effect of *sucCD* and *putA* gene deletion on Hyp, succinate and glutamate production. C. The specific activities of putA and GDH in different recombinant strains. D. Effect of glucose addition on Hyp production and conversion rate with strain *E. coli*Δ*sucCD*Δ*putA*‐*Ds*P4H. AcCoA, acetyl‐CoA; CIT, citrate; *gdh*, glutamate dehydrogenase gene; OAA, oxaloacetate; PEP, phosphoenolpyruvate; *proA*, glutamate‐semialdehyde dehydrogenase gene; *proB*, γ‐glutamyl kinase gene; *proC*, Δ^1^‐pyrroline‐5‐carboxylate reductase gene; PYR, pyruvate; SUCC, succinyl‐CoA.

α‐KG can be supplied through two metabolic pathways, the tricarboxylic acid (TCA) cycle from glucose and l‐proline degradation pathway from l‐proline (Shibasaki *et al.*, 2000b; Fig. 3A). The conversion of α‐KG to succinate in the TCA cycle is sequentially catalysed by α‐KG dehydrogenase complex and succinyl‐CoA synthetase (sucCD) in *E. coli*, and this conversion can also be achieved by one‐step reaction with P4H (Lawrence *et al.*, 1996). Thus, when the *sucC* and *sucD* genes are simultaneously deleted in *E. coli*‐*Ds*P4H, flux partitioning at the α‐KG node may be mainly redirected towards Hyp synthesis under the driving force of P4H that also functions as another bypass route for succinate formation. To demonstrate this idea, the *sucC* and *sucD* genes were simultaneously deleted in *E. coli*‐*Ds*P4H, and the resulting strain *E. coli*Δ*sucCD*‐*Ds*P4H showed a 139.1% increase in Hyp production up to 30.6 g l^−1^ without α‐KG addition compared to that of *E. coli*‐*Ds*P4H, and succinate accumulation was only increased to 5.8 g l^−1^ (Fig. 3B). To our surprise, the concentration of glutamate was up to 10.2 g l^−1^, which was 5.8‐fold higher than that of *E. coli*‐*Ds*P4H (Fig. 3B). To identify sources of glutamate, the activities of proline dehydrogenase (PutA) and glutamate dehydrogenase (GDH) were assayed. PutA activity in *E. coli*Δ*sucCD*‐*Ds*P4H was increased by 37.9% compared to that of *E. coli*‐*Ds*P4H, but GDH activity was kept consistent with that of *E. coli*‐*Ds*P4H (Fig. 3C). These results indicated that glutamate formation was from l‐proline degradation, but not α‐KG.

To reduce l‐proline degradation and convert more l‐proline to Hyp, putA in l‐proline degradation pathway was deleted in *E. coli*Δ*sucCD*‐*Ds*P4H, and its activity in the resulting strain *E. coli*Δ*sucCD*Δ*putA*‐*Ds*P4H was decreased significantly (Fig. 3C). In addition, the formation of glutamate was resulted in a 3.6‐fold decrease compared to that of *E. coli*Δ*sucCD*‐*Ds*P4H (Fig. 3B). Based on this, Hyp production and its conversion rate were up to 41.3 g l^−1^ and 72.5%, both of which were increased by 35.0% compared to that of *E. coli*Δ*sucCD*‐*Ds*P4H (Fig. 3B). In addition, succinate accumulation was only increased to 8.4 g l^−1^ (Fig. 3B). To further improve conversion rate from l‐proline to Hyp without α‐KG addition, glucose addition was optimized to balance flux partitioning at the α‐KG node between cell growth and Hyp production. With the increase of glucose addition from 10 to 30 g l^−1^, Hyp production was increased, and the maximal Hyp titres and conversion rate reached 47.6 g l^−1^ and 83.6% at 30 g l^−1^ glucose (Fig. 3D). When the addition of glucose was over 30 g l^−1^, Hyp production was reduced slightly (Fig. 3D). These abovementioned results demonstrated that *E. coli*Δ*sucCD*Δ*putA*‐*Ds*P4H could efficiently convert l‐proline to Hyp without α‐KG addition. However, Hyp productivity was only 0.66 g l^−1^ h^−1^ that did not meet the needs of industrial application, possibly due to the fact that Hyp production with P4H is a high‐oxygen‐demand process (Zhao *et al.*, 2017).

### Expressing haemoglobin to improve Hyp production

**Fig. 4:**
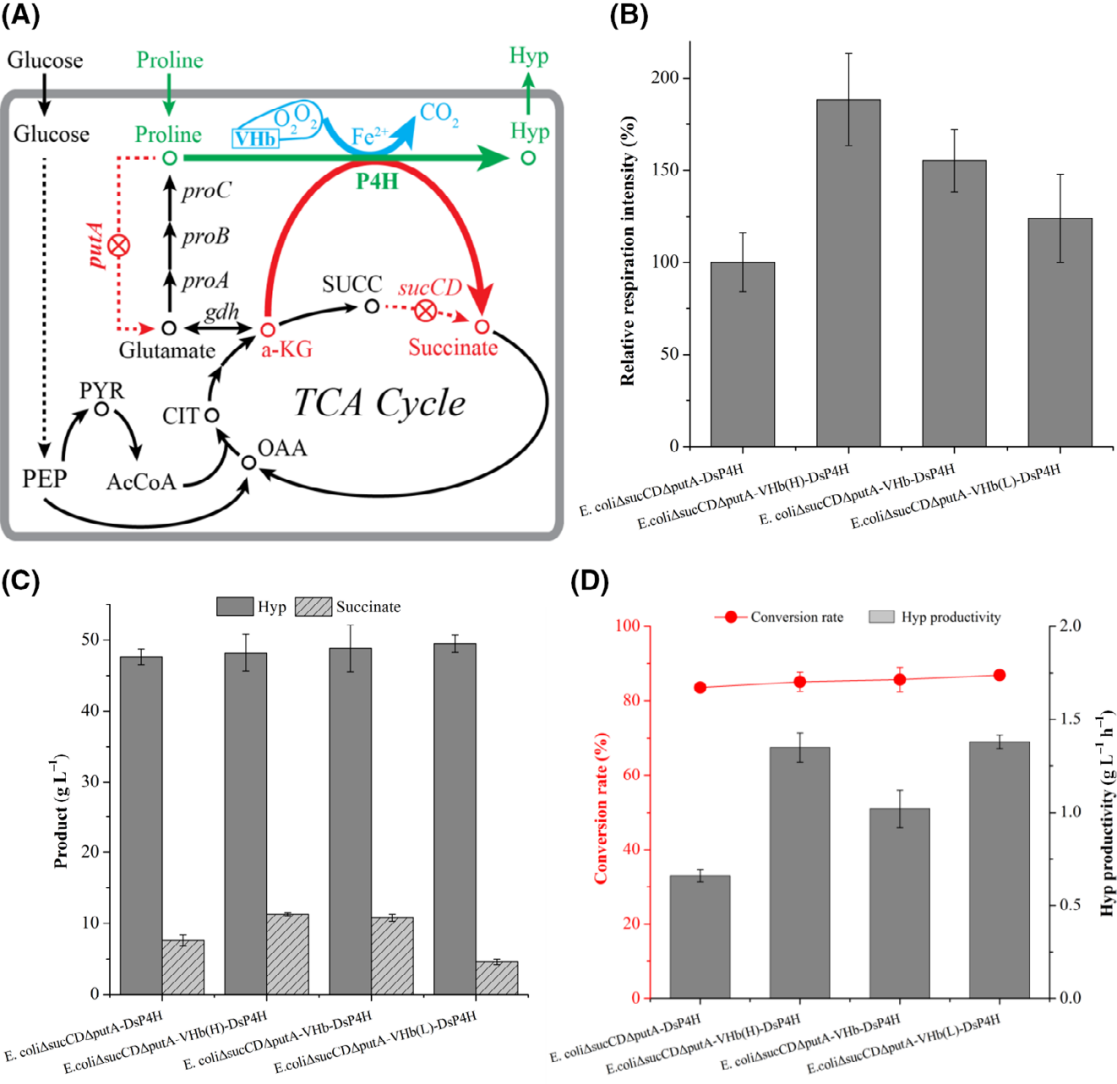
Expressing haemoglobin to improve Hyp production. A. VHb overexpression for oxygen binding in recombinant *E. coli* strain to produce Hyp. B. Effect of different VHb expression strengths on the respiration intensity of *E. coli* strains. C. Effect of different VHb expression strengths on Hyp and succinate production. D. Effect of different VHb expression strengths on Hyp productivity and conversion rate. AcCoA, acetyl‐CoA; CIT, citrate; *gdh*, glutamate dehydrogenase gene; OAA, oxaloacetate; PEP, phosphoenolpyruvate; *proA*, glutamate‐semialdehyde dehydrogenase gene; *proB*, γ‐glutamyl kinase gene; *proC*, Δ^1^‐pyrroline‐5‐carboxylate reductase gene; PYR, pyruvate; SUCC, succinyl‐CoA.

*Vitreoscilla* haemoglobin (VHb) is an oxygen‐binding protein with an oxygen dissociation rate constant of 5600/s (Orii and Webster, 1986), which has been widely used in recombinant strains to improve growth and production of target compounds (Zhang *et al.*, 2013; Akbas *et al.*, 2014; Li and Zhang, 2015). Thus, VHb was overexpressed in *E. coli*Δ*sucCD*Δ*putA*‐*Ds*P4H to enhance cell respiration by promoting oxygen transfer to the intracellular terminal oxidases (Fig. 4A). The respiration intensity of *E. coli*Δ*sucCD*Δ*putA*‐VHb‐*Ds*P4H was increased by 55.2% compared to that of *E. coli*Δ*sucCD*Δ*putA*‐*Ds*P4H (Fig. 4B). Further, Hyp production (48.8 g l^−1^) was only 2.5% higher than that of *E. coli*Δ*sucCD*Δ*putA*‐*Ds*P4H (Fig. 4C), and Hyp productivity (1.02 g l^−1^ h^−1^) was increased by 54.5% (Fig. 4D). However, *E. coli*Δ*sucCD*Δ*putA*‐VHb‐*Ds*P4H still accumulated 10.8 g l^−1^ succinate (Fig. 4C). These results revealed that VHb overexpression tended to balance cell respiration and Hyp production, but this balance should be further optimized.

To realize the optimal balance between cell respiration and Hyp production, the expression of VHb was further improved by ribosome binding sites (RBSs) with different strengths from our previous study (Zhang *et al.*, 2018b). Each RBS was assembled into operons and cloned into plasmids with the same promoter, and then all these combinations could be expressed respectively. With the increase of RBS strengths, the respiration intensity was decreased gradually (Fig. 4B). Further, Hyp production (> 48 g l^−1^) and conversion rate (> 85%) were similar to each other, but Hyp productivity with *E. coli*Δ*sucCD*Δ*putA*‐VHb_(L)_‐*Ds*P4H (1.38 g l^−1^ h^−1^) was increased by 35.3% compared to that of *E. coli*Δ*sucCD*Δ*putA*‐VHb‐*Ds*P4H (Fig. 4C and D). In addition, its succinate accumulation was reduced to 4.6 g l^−1^ (Fig. 4C). These results displayed that the optimization of RBS strengths could improve the balance of cell respiration and Hyp production, thus enhancing Hyp productivity.

### Producing Hyp with *E. coli*ΔsucCDΔputA‐VHb_(L)_‐DsP4H in a 5‐l bioreactor

**Fig. 5:**
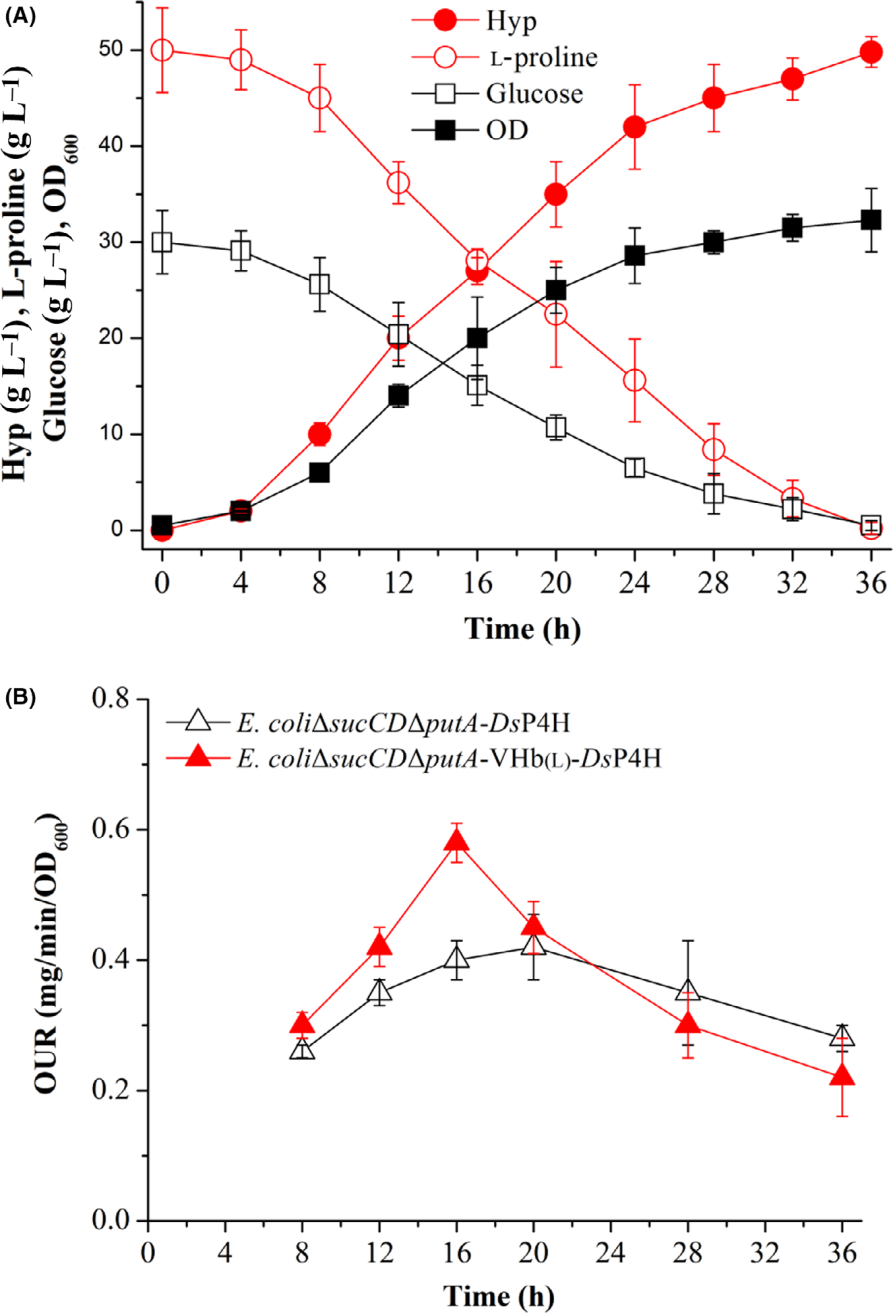
Hyp production with *E. coli*Δ*sucCD*Δ*putA*‐VHb_(L)_‐*Ds*P4H in a 5‐l bioreactor. A. Hyp, l‐proline, glucose and OD. B. Comparison of OUR between *E. coli*Δ*sucCD*Δ*putA*‐VHb_(L)_‐*Ds*P4H and *E. coli*Δ*sucCD*Δ*putA*‐*Ds*P4H.

Based on the above experiments, we further explored the potential of using whole‐cell biocatalyst of the recombinant strain *E. coli*Δ*sucCD*Δ*putA*‐VHb_(L)_‐*Ds*P4H for the transformation of l‐proline to Hyp in 5‐l bioreactors. In this batch culture, glucose and l‐proline were rapidly consumed during cell growth and Hyp synthesis and were depleted completely at 36 h (Fig. 5A). In addition, strain *E. coli*Δ*sucCD*Δ*putA*‐VHb_(L)_‐*Ds*P4H grew continuously from 0 to 36 h, and obtained a maximal OD of 32.3 (Fig. 5A). Hyp accumulated gradually in the broth from 0 to 36 h, and the final Hyp titre, conversion rate and productivity were up to 49.8 g l^−1^, 87.4% and 1.38 g l^−1^ h^−1^ respectively (Fig. 5A). Further, the highest oxygen uptake rate (OUR) of *E. coli*Δ*sucCD*Δ*putA*‐VHb_(L)_‐*Ds*P4H was 38.1% higher than that of *E. coli*Δ*sucCD*Δ*putA*‐*Ds*P4H (Fig. 5B), due to VHb overexpression. These results indicated that *E. coli*Δ*sucCD*Δ*putA*‐VHb_(L)_‐*Ds*P4H was stable for scale‐up culture, suggesting that it has great potential for industrial production of Hyp in the future.

## Discussion

Hyp production can be catalysed by proline‐4‐hydroxylase (P4H) to hydroxylate L‐proline with α‐ketoglutarate (α‐KG) and oxygen as co‐substrates to generate succinate and carbon dioxide (CO_2_) in the presence of ferrous ion. In this study, to improve the catalytic performance of *E. coli*, chassis engineering was used to enhance α‐KG supply by rewiring the TCA cycle and l‐proline degradation pathway and promote oxygen transfer by fine‐tuning heterologous haemoglobin expression. These strategies resulted in a significant increase in Hyp titre, conversion rate and productivity up to 49.8 g l^−1^, 87.4% and 1.38 g l^−1^ h^−1^ respectively. These results lay a good foundation for industrial production of Hyp in the future and pave the way to the development of whole‐cell biocatalysis through combining metabolic engineering with enzymatic transformation for microbial production of other chemicals.

Metabolic engineering plays an important role in improving the catalytic efficiency of whole‐cell biocatalyst through modifying cellular metabolic network to overcome potential metabolic bottlenecks. Interconnection between cellular metabolism and enzymatic transformation can be bridged tightly by co‐substrates or redox cofactors required for enzyme activity (Blank *et al.*, 2010; Falcioni *et al.*, 2013; Schrewe *et al.*, 2013; Theodosiou *et al.*, 2015). As a typical example, Hyp biosynthesis from l‐proline can be catalysed by P4H with α‐KG and oxygen as co‐substrates to generate succinate and CO_2_ in the presence of ferrous ion. α‐KG can be supplied to P4H from the TCA cycle and L‐proline degradation pathway, and in turn central carbon metabolism can assimilate and recycle the coproduct of this enzymatic reaction succinate (Theodosiou *et al.*, 2017). Generally, the availability of l‐proline and α‐KG in this hydroxylation will be influenced through five pathways (Zhang *et al.*, 2018a): (i) degradation of l‐proline with PutA (Falcioni *et al.*, 2013), (ii) biosynthesis of l‐proline from glutamate caused by GDH and proBAC, (iii) oxidation of citrate via the TCA cycle to form α‐KG, followed by oxidation of α‐KG to succinate with SucAB and SucCD, (iv) glyoxylate pathway from isocitrate to succinate via AceA, and (v) phosphorylation of isocitrate dehydrogenase under the action of AceK (Smirnov *et al.*, 2010). An effective method to improve the conversion of l‐proline is to reinforce l‐proline biosynthesis pathway by expressing *proBAC* genes (Zhang *et al.*, 2018a) and interrupt l‐proline degradation pathway by knocking out *putA* gene (Theodosiou *et al.*, 2015) and the TCA cycle by deleting *SucAB* and *SucCD* genes (Zhang *et al.*, 2018a, 2019). In this study, *sucCD* and *putA* genes were deleted in *E. coli*‐*Ds*P4H, and the final engineered strain *E. coli*Δ*sucCD*Δ*putA*‐*Ds*P4H could efficiently convert l‐proline to Hyp without α‐KG addition. The reason is that overexpression of *Ds*P4H in *E. coli* drive the redirection of carbon flux partitioning at the α‐KG node towards the Hyp biosynthesis as another bypass route for succinate formation. In addition, *SucAB* deletion could impair cell growth (Theodosiou *et al.*, 2017; Zhang *et al.*, 2018a), but these strategies in this study had no significant impact on cell growth due to the formation of succinyl‐CoA via SucAB and succinate via *Ds*P4H.

Oxygen transfer is generally regarded as one of the main limiting factors in oxygen‐demand process. P4H catalyses hydroxylation of proline to form Hyp with the co‐substrate of α‐KG undergoing oxidative decarboxylation to succinate. Due to the catalytic characteristics of P4H, Hyp production is a high‐oxygen‐demand process. In addition, Hyp fermentation broths also exhibit high viscosity during culture, which can further hinder oxygen transfer (Shibasaki *et al.*, 1999; Falcioni *et al.*, 2015). During microbial fermentation, oxygen transfer can be generally improved by increasing agitation and aeration rates (Falcioni *et al.*, 2015), but this will cause high energy consumption and result in physical damage to cells (Smith *et al.*, 1990). To overcome this issue, *Vitreoscilla* haemoglobin (VHb), an oxygen‐binding protein (Orii and Webster, 1986), provides a good choice, which can enhance respiration and energy metabolism by promoting oxygen transfer to the intracellular terminal oxidases (Dikshit *et al.*, 1992; Chi *et al.*, 2009). VHb has been widely used to improve cell growth and chemical production such as Hyp (Zhao *et al.*, 2017), poly(ε‐l‐lysine; Xu *et al.*, 2015), polysaccharide (Li *et al.*, 2016), natamycin (Wang *et al.*, 2014) and fatty acids (Suen *et al.*, 2014). In this study, VHb expression in recombinant *E. coli*Δ*sucCD*Δ*putA*‐*Ds*P4H improved Hyp production, presumably by enhancing oxygen transfer. On the one hand, VHb expression enhances the level of dissolved oxygen, thus weakening the anaerobic fermentation pathway and enhancing the aerobic fermentation pathway (Clark, 1989). On the other hand, VHb expression improves cell growth and extends the period of exponential growth in both shake flasks and bioreactor fermentation (Zhang *et al.*, 2013). Third, succinate is the major by‐product resulting from hydroxylation of proline with P4H, and VHb expression was able to reduce the concentration of succinate in this study, suggesting that the VHb‐mediated improvement of oxygen transfer helps the TCA cycle to match up with proline hydroxylation with P4H in *E. coli*Δ*sucCD*Δ*putA*‐VHb_(L)_‐*Ds*P4H. This strategy provides new insights into engineering *E. coli* host by flux coupling for the enzymatic production of Hyp and its related high‐value‐added products.

## Experimental procedures

### Strains and plasmids

*E. coli* F0901 was constructed to produce pyruvate, α‐ketoglutarate (α‐KG) and l‐malate (Dong *et al.*, 2017). The engineered *E. coli* strains used for *trans*‐4‐hydroxy‐l‐proline (Hyp) production in this study were derived from *E. coli* F0901, in which lactate dehydrogenase (*ldhA*), pyruvate oxidase (*poxB*), pyruvate formate lyase (*pflB*), phosphotransacetylase (*pta*), acetate kinase A (*ackA*), fumarate reductase (*frdBC*) and fumarase (*fumB* and *fumAC*) genes were all deleted (Dong *et al.*, 2017). *E. coli* JM109 and plasmid pETM6R1 (Zhang *et al.*, 2018b) were used for plasmid construction. All strains and plasmids used in this study are listed in Table 2.

**Table 2:** Strains and plasmids used in this study

Strains and plasmids	Relevant characteristics	References
Strains		
*E. coli* W3110	F^−^λ^−^rph‐1 INV(rrnD, rrnE)	CGSC
*E. coli* F0901	*E. coli* W3110 △*ldhA*△*poxB*△*pflB*△*pta‐ackA*△*frdBC*△*fumB*△*fumAC*	Dong *et al.* (2017)
*E. coli*‐*Ds*P4H	*E. coli* F0901(pETM6R1‐*Ds*P4H)	This study
*E. coli*‐*Bm*P4H	*E. coli* F0901(pETM6R1‐*Bm*P4H)	This study
*E. coli*‐*Ao*P4H	*E. coli* F0901(pETM6R1‐*Ao*P4H)	This study
*E. coli*‐*Af*P4H	*E. coli* F0901(pETM6R1‐*Af*P4H)	This study
*E. coli*Δ*sucCD*‐*Ds*P4H	*E. coli* F0901Δ*sucCD* (pETM6R1‐*Ds*P4H)	This study
*E. coli*Δ*sucCD*Δ*putA*‐*Ds*P4H	*E. coli* F0901Δ*sucCD*Δ*putA* (pETM6R1‐*Ds*P4H)	This study
*E. coli*Δ*sucCD*Δ*putA*‐VHb_(L)_‐*Ds*P4H	*E. coli* F0901Δ*sucCD*Δ*putA* (pETM6R1‐*Ds*P4H‐VHb_(L)_)	This study
*E. coli*Δ*sucCD*Δ*putA*‐VHb‐*Ds*P4H	*E. coli* F0901Δ*sucCD*Δ*putA* (pETM6R1‐*Ds*P4H‐VHb_(M)_)	This study
*E. coli*Δ*sucCD*Δ*putA*‐VHb_(H)_‐*Ds*P4H	*E. coli* F0901Δ*sucCD*Δ*putA* (pETM6R1‐*Ds*P4H‐VHb_(H)_)	This study
Plasmids		
pKD3	R6Kγ ori, Cm^R^, rgnB(Ter)	Invitrogen
pKD4	R6Kγ ori, Km^R^, rgnB(Ter)	Invitrogen
pKD46	R101 ori, Amp^R^, araBp‐gam‐bet‐exo, repA101(ts)	Invitrogen
pCP20	Amp^R^, Cm^R^, FLP recombinance	Invitrogen
pETM6R1	ColE1 ori, Amp^R^, P_Trc_	Zhang *et al.* (2018b)
pETM6R1‐*Ds*P4H	ColE1 ori, Amp^R^, P_Trc_‐*Ds*P4H	This study
pETM6R1‐*Bm*P4H	ColE1 ori, Amp^R^, P_Trc_‐*Bm*P4H	This study
pETM6R1‐*Ao*P4H	ColE1 ori, Amp^R^, P_Trc_‐*Ao*P4H	This study
pETM6R1‐*Af*P4H	ColE1 ori, Amp^R^, P_Trc_‐*Af*P4H	This study
pETM6R1‐*Ds*P4H‐VHb_(L)_	ColE1 ori, Amp^R^, P_Trc_‐*Ds*P4H_‐rbs03_‐VHb_(L)_	This study
pETM6R1‐*Ds*P4H‐VHb_(M)_	ColE1 ori, Amp^R^, P_Trc_‐*Ds*P4H_‐rbs09_‐VHb_(M)_	This study
pETM6R1‐*Ds*P4H‐VHb_(H)_	ColE1 ori, Amp^R^, P_Trc_‐*Ds*P4H_‐rbs10_‐VHb_(H)_	This study

### DNA manipulation

Standard molecular cloning was used for plasmid construction according to the protocol of ePathBrick Vectors Assembly (Xu *et al.*, 2012). Proline 4‐hydroxylase (P4H) gene from *Dactylosporangium sp*. RH1 (*Ds*P4H, Gene ID: D78338.1) was artificially synthesized with codon optimization by Shanghai Sunny Biotechnology. P4H gene from *Bacillus megaterium* WSH‐002 (*Bm*P4H, Gene ID: BMWSH_2348) was amplified from the corresponding chromosomal DNA by PCR. *Ao*P4H (Gene ID: AOR_1_1350154) and *Af*P4H (Gene ID: AFLA_030540) genes were amplified by PCR using the *c*DNA of *Aspergillus oryzae* RIB40 and *Aspergillus flavus* NRRL3357 as a template respectively. Haemoglobin gene from *Vitreoscilla sp*. HG1 (VHb, Gene ID: AF292694.1) was artificially synthesized by Shanghai Sunny Biotechnology. All gene deletions were performed according to the classical red homologous recombination method (Datsenko and Wanner, 2000). The expression of VHb was optimized by ribosome binding sites (RBSs) with different strengths (RBS03: CGACATAACGTTAGAAAAGAATAAGGTAGTTTC; RBS09: TATTTAAACTATCACGACATAAGGAGGTCAGGG; RBS10: AAGAGGGCGCGGCAGAGAAGGAGGAGGTAAGAA) from our previous study (Zhang *et al.*, 2018b).

### Media

Medium Luria–Bertani (LB) used for seed cultures: 5 g l^−1^ yeast extract, 10 g l^−1^ peptone, 5 g l^−1^ NaCl. Ampicillin (100 mg ml^−1^) was added appropriately when needed.

Modified AM1 mineral salts medium A used for fermentation in shake flasks: 50 g l^−1^ l‐proline, 10 g l^−1^ α‐KG, 20 g l^−1^ glucose, 20 g l^−1^ tryptone, 10 g l^−1^ yeast extract, 2.63 g l^−1^ (NH_4_)_2_HPO_4_‐12H_2_O, 0.87 g l^−1^ NH_4_H_2_PO_4_, 0.15 g l^−1^ KCl, 0.3 g l^−1^ FeSO_4_, 0.37 g l^−1^ MgSO_4_‐7H_2_O, and 1 ml trace element solution (2.4 g l^−1^ FeCl_3_‐6H_2_O, 0.3 g l^−1^ CoCl_2_‐6H_2_O, 0.3 g l^−1^ CuCl_2_, 0.3 g l^−1^ ZnCl_2_‐4H_2_O, 0.3 g l^−1^ NaMnO_4_, 0.075 g l^−1^ H_3_BO_3_, 0.5 g l^−1^ MnCl_2_‐4H_2_O, dissolve in 0.12 M HCl). Ampicillin (100 mg ml^−1^) and IPTG (0.4 mmol l^−1^) were added appropriately when needed.

Modified AM1 mineral salts medium B used for fermentation in a 5‐l bioreactor: 50 g l^−1^ l‐proline, 30 g l^−1^ glucose, 20 g l^−1^ tryptone, 10 g l^−1^ yeast extract, 2.63 g l^−1^ (NH_4_)_2_HPO_4_‐12H_2_O, 0.87 g l^−1^ NH_4_H_2_PO_4_, 0.15 g l^−1^ KCl, 0.3 g l^−1^ FeSO_4_, 0.37 g l^−1^ MgSO_4_‐7H_2_O, and 1 ml trace element solution (2.4 g l^−1^ FeCl_3_‐6H_2_O, 0.3 g l^−1^ CoCl_2_‐6H_2_O, 0.3 g l^−1^ CuCl_2_, 0.3 g l^−1^ ZnCl_2_‐4H_2_O, 0.3 g l^−1^ NaMnO_4_, 0.075 g l^−1^ H_3_BO_3_, 0.5 g l^−1^ MnCl_2_‐4H_2_O, dissolve in 0.12 M HCl). Ampicillin (100 mg m l^−1^) and IPTG (0.4 mmol l^−1^) were added appropriately when needed.

### Culture conditions

The seed culture inoculated from a slant was cultivated on a reciprocal shaker (200 rpm) at 37°C in a 250‐ml flask containing 25 ml medium LB for 12 h. The broth was centrifuged, and then, the supernatant liquid was discarded. Next, the pellet was suspended in demineralized medium A. After that, the cell suspension was divided equally between 500‐ml flasks containing 50 ml fresh medium A with an initial biomass *A*_600_ of 0.5. The medium was buffered by adding 50 g l^−1^ CaCO_3_ followed by fermentation at 37°C for 72 h with rotation at 200 rpm.

Batch fermentation was performed in a 5‐l bioreactor (NBS) containing 2.5 l medium B with an initial biomass *A*_600_ of 0.5. Enzyme expression was induced when biomass reached an *A*_600_ of 2.0 by adding 0.4 mmol l^−1^ IPTG. Fermentation was performed at 37°C for 36 h with agitation speed at 200 rpm and aeration rate at 1.0 vvm. Culture pH was controlled at 6.5 using 25% NH_4_OH.

### Analytical methods

The optical density at 600 nm was measured using a spectrophotometer. Glucose concentration was quantified by a biosensor SBA‐90 (Dong *et al.*, 2017). The glutamate, succinate and α‐KG concentrations were determined by high‐performance liquid chromatography (HPLC; Zhang *et al.*, 2009). l‐proline and Hyp were assayed by HPLC with a Zorbax Eclipse XDB‐C_18_ column (Agilent, Santa Clara, CA, USA) at 40°C after derivatization with 2,4‐dinitrofluorobenzene (Zhang *et al.*, 2019).

### Measurement of conversion rate

Conversion rate was determined using the following equation:

$$\text{Conversion}\;\text{rate}\;\left(\%\right)=\frac{M3}{M1-M2}\times 100$$

where *M*1 is the concentration of l‐proline before conversion, *M*2 is the remaining concentration of l‐proline after conversion, and *M*3 is the concentration of l‐proline used for Hyp production.

### Measurement of oxygen consumption rate

Oxygen consumption rate measurements were performed as described by Srikumar *et al.* (2013) with minor modifications. *E. coli* cells were cultured at 37°C in medium A until they reached the exponential growth phase. Then, cultures were collected, washed with 50 mM potassium phosphate buffer (pH 6.8), and resuspended to OD_600_ = 0.1. Resuspended cells were used to seed XF 96‐well microplates (Seahorse Biosciences, Santa Clara, CA, USA). Plates were centrifuged at 2000 rpm for 2 min and then allowed to rest for 30 min at 37°C. Oxygen consumption rate was measured according to the manufacturer’s manual on a Seahorse XF96 Analyzer. XF96 culture plates and the corresponding sensor cartridge were placed in Seahorse instrument, and temperature was maintained at 37°C. To equilibrate culture to instrument conditions, an initial wait time (20 min) was added. To allow for cell settling, a wait time (1 min) was also included after mixing 1 min. Before adding azide to a final concentration of 0.05% in media, three measurements were taken for the basal reading. Then, three additional readings were taken. The mean of the three readings across the span of 2 min was calculated for each well.

### Measurement of oxygen uptake rate

Oxygen uptake rate (OUR) was determined in a 5‐l bioreactor using the dynamic gassing‐out method as described by Bhave and Chattoo (2003), Chen *et al.* (2007), and Wu *et al.* (2018). The airflow to bioreactor was stopped with simultaneous reduction in agitation rate to 100 rpm, and dissolved oxygen (DO) was measured by DO electrode (Mettler, Columbus, OH, USA ). Aeration and agitation were restored before DO values reached critically low values. Time course of DO decrease was recorded, and the slope of DO vs time plots was used to determine OUR. 100% DO saturation indicated an oxygen concentration of 8 mg l^−1^ (Bhave and Chattoo, 2003).

### Enzymes activity assays

P4H activity was measured as reported by Yi *et al.* (2014). The reaction mixture containing 80 mM MES buffer (pH 6.5), 4 mM l‐proline, 8 mM α‐KG, 2 mM FeSO_4_, 4 mM l‐ascorbic acid, and cells were incubated at 35°C for 10 min with shaking, and then, cellular activity was inactivated completely by heat treatment at 100°C for 5 min. The concentration of Hyp in this mixture was determined after centrifugation. One unit of P4H activity was defined as the amount of enzyme that forms 1 nmol of Hyp in one minute.

Proline dehydrogenase (PutA) activity was determined in toluene‐treated whole cells as described by Deutch *et al.* (1985) with minor modifications. The reaction mixture containing toluene‐treated cells, l‐proline (1.0 M), *o*‐aminobenzaldehyde (50 mM) and ethanol (20%) was incubated at 37°C for 2 h with vigorous shaking, and then, this assay was terminated by adding trichloracetic acid (20%). The concentration of Δ1‐pyrroline‐5‐carboxylate in this mixture was measured to calculate PutA activity.

Glutamate dehydrogenase (GDH) activity was assayed by measuring spectrophotometrically the oxidation of NADPH at 340 nm at 22°C (Veronese *et al.*, 1975). The reaction system containing 0.1 M Tris‐HCl buffer (pH 8.0), 0.1 M NH_4_Cl, 2.5 mM α‐KG and 0.1 mM NADPH was initiated by adding cell extracts. One unit of GDH activity was defined as the amount of enzyme that converts 1 pmol of NADPH in one minute.

Protein concentrations in cell extracts were determined by the Lowry method using bovine serum albumin as the standard (Lowry *et al.*, 1951). The specific enzyme activity was defined as the enzyme activity per milligram of protein under the assay conditions.

## Conflict of interest

The authors declare no conflict of interest.
